# High expression of cuproptosis-related gene FDX1 in relation to good prognosis and immune cells infiltration in colon adenocarcinoma (COAD)

**DOI:** 10.1007/s00432-022-04382-7

**Published:** 2022-09-29

**Authors:** Lizong Wang, Yi Cao, Wei Guo, Jingyun Xu

**Affiliations:** 1 General Practice Department, The First Affiliated Hospital of Wannan Medical College (Yijishan Hospital of Wannan Medical College), Wuhu, Anhui Province China; 2 School of Basic Medicine, Wannan Medical College, NO. 22 Wenchang west road, Wuhu, Anhui Province China

**Keywords:** FDX1, Cuproptosis, Prognosis, Immune infiltrate, COAD

## Abstract

**Background:**

Cuproptosis induced by FDX1 is a newly discovered mechanism regulating cell death. However, the role of FDX1 in the pathogenesis of colon adenocarcinoma (COAD) remains to be studied.

**Methods:**

FDX1 expression was analyzed with The Cancer Genome Atlas (TCGA) database and Human Protein Atlas (HPA) database. Association between FDX1 expression and COAD prognosis was investigated via the Kaplan–Meier (KM) survival curve. The differentially expressed genes (DEGs) of FDX1 were screened with R packages and the PPI were constructed via STRING database. Cytoscape software was used to detect the most profound modules in the PPIs network. CancerSEA database was used to analyze the effect of FDX1 expression levels on different functional status of COAD cells. The relationship between FDX1 expression and immune infiltration of COAD was analyzed by TIMER2.0 database. The COAD patients with high expression of FDX1 by Western blot, and the levels of immune infiltration were measured by flow cytometry.

**Results:**

FDX1 was low expressed in most cancers, such as BRCA, KICH, and COAD. The overall survival (OS) and disease-specific survival (DSS) of COAD with high FDX1 expression were better than that of the low expression group. GO-KEGG enrichment analysis revealed that FDX1 and its co-expressed genes played an important role in the pathogenesis of COAD. Moreover, FDX1 expression in COAD were positively associated with “quiescence” and “inflammation” but negatively correlated with “invasion”. FDX1 expression was positively correlated with infiltration levels of CD8^+^ T cells, NK cells, and neutrophils. Oppositely, FDX1 expression was negatively correlated with that of CD4^+^ T cells and cancer-associated fibroblasts (CAFs). Finally, 6 COAD patients with high expression of FDX1 were screened, and the proportion of CD8^+^ T cells in cancer tissues of these patients was significantly higher than that in paracancerous, while the CD4^+^ T cells presented the opposite pattern.

**Conclusion:**

FDX1 plays a role in inducing cuproptosis and modulating tumor immunity, which could be considered as potential therapeutic targets in COAD.

## Introduction

Colon adenocarcinoma (COAD) is the third common malignancy worldwide and the second common cause of cancer-related death (Haraldsdottir et al. 2014; Torre et al. 2015; Baidoun et al. 2021). COAD has high mortality and morbidity, with more than 1 million new cases each year, as it is closely associated with lymph node metastasis (Brenner et al. 2014; Chen et al. 2016). Despite steady progress in screening, early diagnosis (e.g., colonoscopy), and treatment (e.g., chemotherapy, surgical tumor resection, and adjuvant therapy) for the disease in recent decades (Kuipers et al. 2015; Brown and Solomon 2018; Matsuda et al. 2018), however, the prognosis of patients with COAD remains poor due to advanced stage of diagnosis and the high frequency of metastasis and recurrence. The 5-year survival rate of patients with colorectal cancer metastasis is still less than 10% (Pizzini et al. 2013), and becoming a major worldwide health problem. Therefore, the development of novel potential biomarkers for prognostic prediction and therapeutic intervention may lead to better treatment strategies for COAD patients.

Cuproptosis is a newly discovered mechanism that differs from all regulatory mechanisms of cell death that have known, including apoptosis, ferroptosis, pyroptosis, and necrotizing apoptosis. Main mechanism of cuproptosis is copper ion that targets the tricarboxylic acid circulating protein lipoylated and aggregation, subsequent loss of iron–sulfur cluster proteins, which leads to protein toxic stress and ultimately cell death (Tsvetkov et al. 2022). FDX1 is a key regulator of cell death induced by copper ionophore and an upstream regulator of protein lipoylation; FDX1 knockout results in a complete loss of protein lipoylation, shielding cells from copper toxicity (Tsvetkov et al. 2022). However, the clinical impacts of cuproptosis-related genes FDX1 in COAD remain largely unclear.

In this study, bioinformatics methods were used to analyze the expression level of FDX1 and its related genes, differentially expressed genes and their functions in colon cancer. Meanwhile, the correlations between FDX1 expression and survival rate and its effect on different functional states of cancer cells were also analyzed. Finally, the effect of FDX1 expression on the tumor microenvironment was predicted, and clinical samples were examined by flow cytometry to confirm our prediction. Based on the findings described above, high expression of FDX1 improves survival in COAD patients, which may be related to the regulation of FDX1 in different functional states of tumor cells and immune microenvironment.

## Materials and methods

### Selection of samples and ethics

This study involved clinical samples collected in the First Affiliated Hospital of Wannan Medical College and pathologically diagnosed with colon cancer. No treatment was received prior to surgery. The adjacent normal tissues were collected > 3 cm from the tumor margin. This study was approved by the Ethics Committee of Wannan Medical College (NO. 2022081).

### FDX1 expression analysis in TCGA database and HPA database

Samples of pan-cancer and colon cancer were selected from the TCGA database (https://portal.gdc.cancer.gov/) for the analysis of FDX1 expression in tumor and normal tissues. Use ggplot2 (version 3.3.3) to plot or visualize the results. FDX1 protein immunohistochemistry in human normal tissues and tumor tissues can also be obtained from the Human Protein Atlas (HPA, https://www.proteinatlas.org/) database.

### Association between FDX1 expression and COAD survival prognosis

Overall survival (OS) data and disease-specific survival (DSS) data were downloaded from TCGA database (https://portal.gdc.cancer.gov/) COAD project. The Kaplan–Meier (KM) survival curve analysis is implemented by R software package “Survival” (version 3.2–10) and “Survminer” (Visualization, version 0.4.9). Cox regression or logrank test was used to analyze the relationship between FDX1 expression and the survival rate of COAD patients, as well as effects of FDX1 expression on prognosis in different clinical variables.

### Screening and analysis of FDX1’s differentially expressed genes and related genes

Differentially expressed genes (DEGs) and functionally related genes of FDX1 in COAD were screened from TCGA database using R packages DESeq2 (version 1.26.0) and stat (version 3.6.3). GO-KEGG enrichment analysis was performed by Bioconductor package ‘‘clusterProfiler’’. Enriched gene sets with *P* value < 0.05 and FDR < 0.25 were considered statistically significant. In addition, protein–protein interaction (PPI) network between PDX1 and its associated genes was constructed by a STARING tool (v11.5, https://www.string-db.org/). The confidence score > 0.7 was considered significant. PPIs were analyzed via Cytoscape software, and MCODE plugin was used to detect the most profound modules from the PPIs network.

### Single cell sequencing data analysis

CancerSEA (http://biocc.hrbmu.edu.cn/CancerSEA/home.jsp) was used to analyze the effect of FDX1 expression levels on different functional status of cancer cells at the single cell level.

### Immune infiltration analysis

The relationship between FDX1 expression and immune infiltration of COAD was analyzed by TIMER2.0 website (http://timer.comp-genomics.org/), as well as the correlation between FDX1 expression and cancer-associated fibroblast (CAFs) of COAD was explored.

### Screening of clinical samples

Total protein of the samples was extracted and the protein concentration was detected. Then, the colon cancer patients with high expression of FDX1 were screened by Western Blot. The primary antibodies used were as follows: FDX1 antibody (abcam, ab108257) (diluted at 1:5000), GAPDH recombinant antibody (proteintech, 80570–1-RR) (diluted at 1:5000), HRP-labeled goat anti-rabbit IgG (Beyotime, A0208) (diluted at 1:1000). Finally, the signal was detected by chemiluminescence, and the bands’ gray value was measured by ImageJ software.

### Flow cytometry

Cell suspensions were prepared, respectively. Reference-based methods (Tian et al. 2018), the proportion of CD4^+^ T cells and CD8^+^ T cells in cancer and paracancerous were investigated by flow cytometer (Beckman Coulter, FC 500 MPL) and analyzed by FlowJo software. The primary antibodies used were as follows: CD3-PC5, CD4-PE, and CD8-ECD (Beckman Coulter, NO. 6607013).

## Results

### The mRNA expression analysis of FDX1

**Fig. 1:**
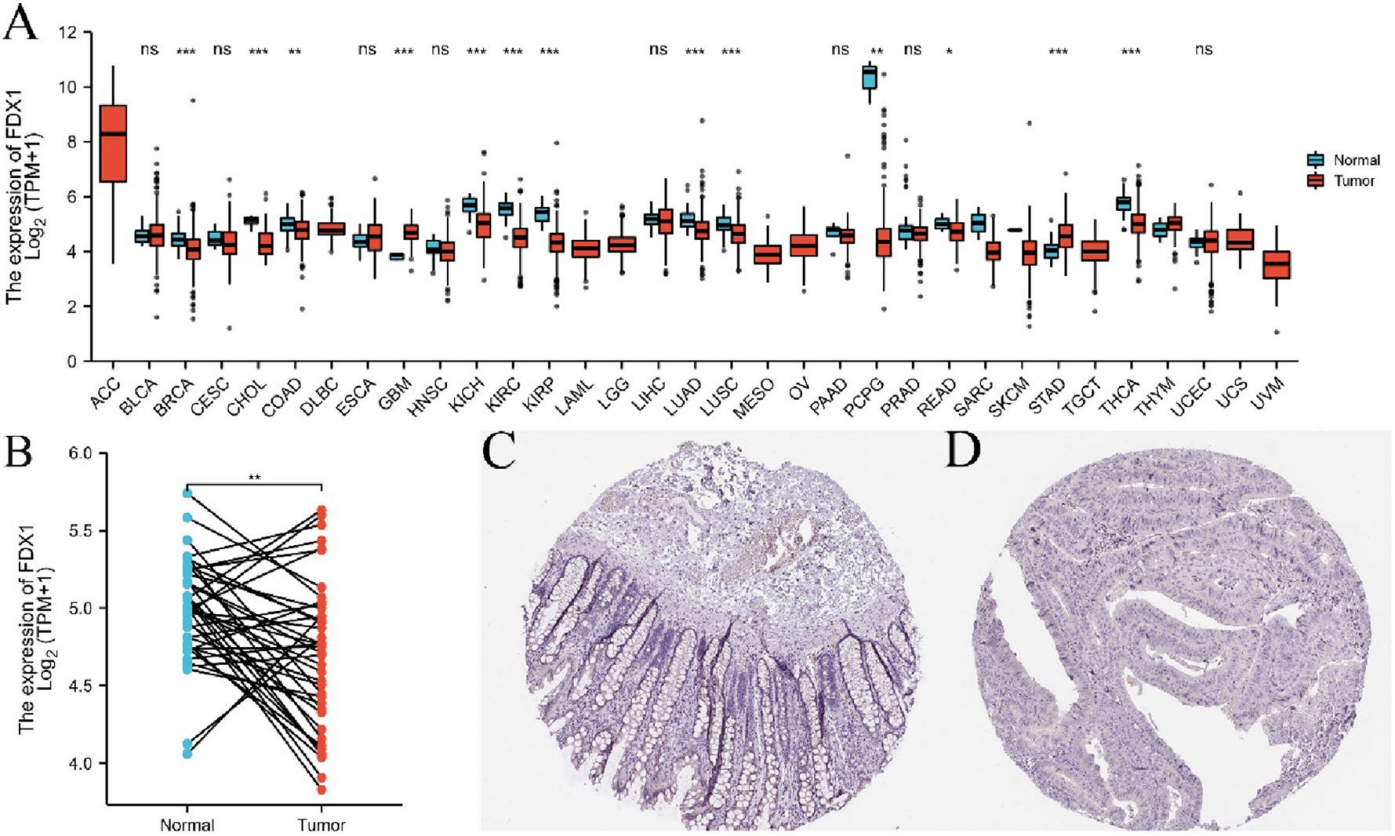
FDX1 expression levels in pan-cancer. **A** Expression of FDX1 in distinct cancers compared with normal tissues. **B** Expression of FDX1 in COAD compared with normal tissues, *N* = 41 (paired samples). **C** The level of FDX1 protein in normal tissue in the human protein atlas. **D** The level of FDX1 protein in COAD tissue in the Human Protein Atlas. *P < 0.05, **P < 0.01, ***P < 0.001, ns: no statistically significant

Expression levels of FDX1 in 33 cancers were analyzed from the TCGA database. The result revealed that FDX1 was low expressed in most cancers, such as BRCA, KICH, KIRC, LUAD, LUSC, PCPG, and THCA (Fig. 1A); as well as COAD (Fig. 1B). Immunohistochemical results in HPA database showed that FDX1 was expressed in mucosa epithelium, lamina propria and submucosa of normal colon tissue (Fig. 1C), but no obvious expression was observed in colon cancer tissue (Fig. 1D).

### Association between FDX1 expression and survival

**Fig. 2:**
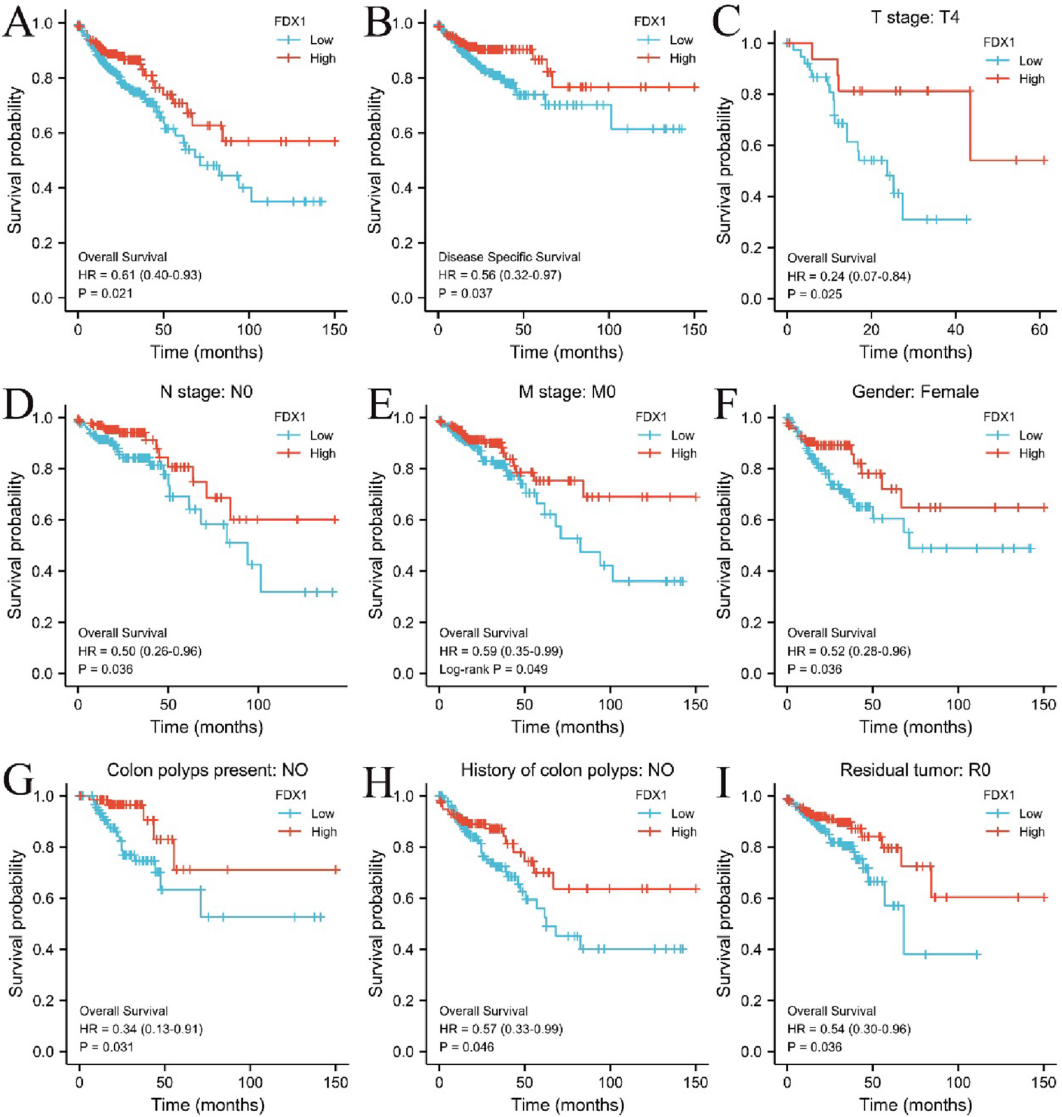
Prognostic value of mRNA level of FDX1 in patients with COAD (Kaplan–Meier plotter). **A**, **B** The correlations between FDX1 and OS [*N* (low/high) = 297/180] or DSS [*N* (low/ high) = 287/174] of COAD patients were evaluated by TCGA database. (**C**–**I**) The correlations between FDX1 and OS of COAD patients were evaluated in different clinical variables

According to the Kaplan–Meier survival curves analysis, COAD patients with higher FDX1 expression showed that overall survival (OS) and disease-specific survival (DSS) were higher than that of the low expression group (*P* < 0.05, Fig. 2A, B). Moreover, high expression of FDX1 also has a positive effect on prognosis (OS) in different clinical variables, such as T4 stage, N0 stage, M0 stage, female, no colon polyps present, no history of colon polyps and R0 residual tumor (*P* < 0.05, Fig. 2C–I).

### Genes analysis of closely associated with FDX1 in COAD

**Fig. 3:**
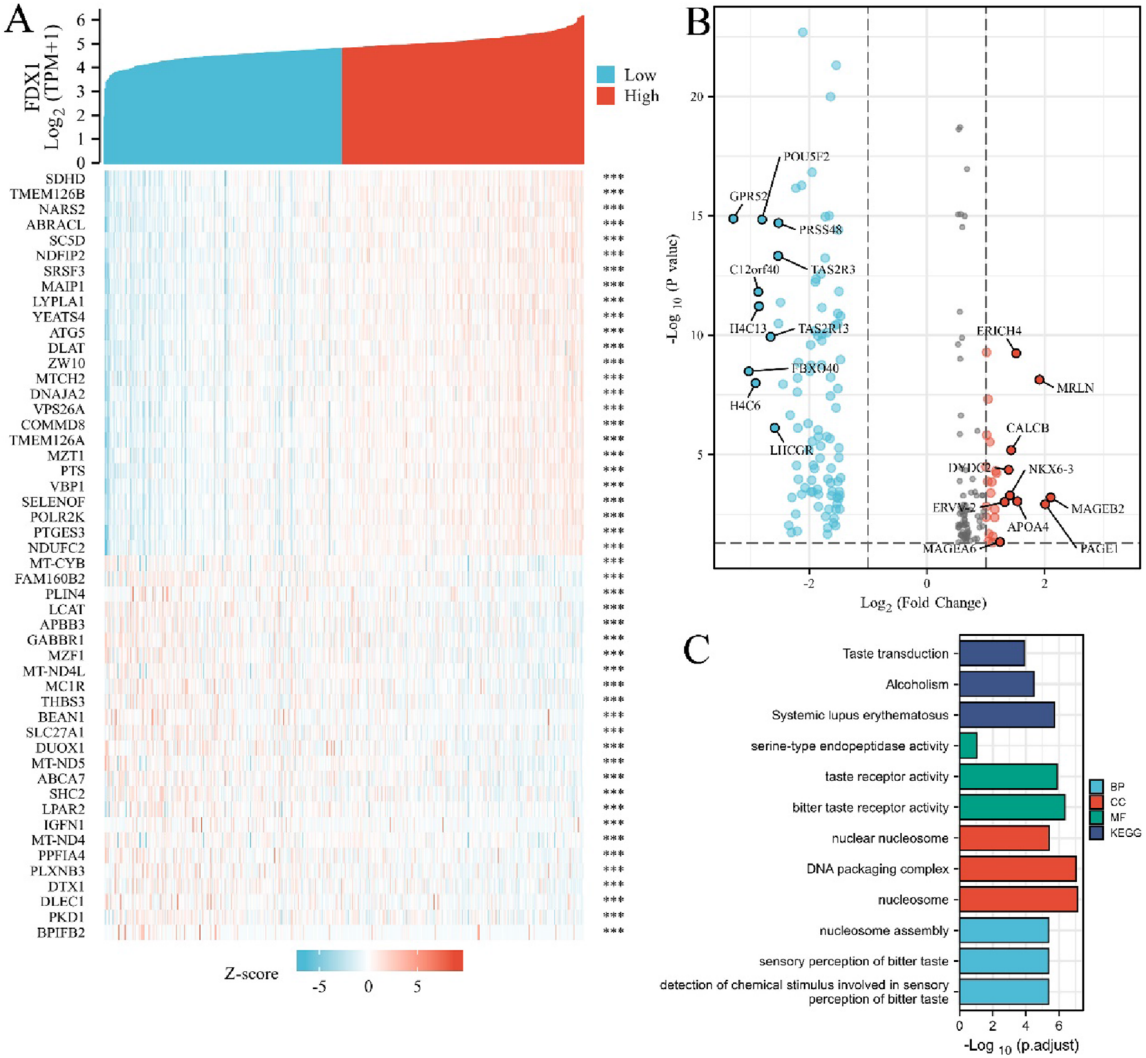
Screening and functional analysis of genes associated with FDX1 in COAD. **A** The top 25 genes of positively or negatively correlated with FDX1 in COAD were showed in Heatmap. **B** Volcano map of all DEGs in COAD and the control group analyzed. The top 10 up-regulated or down-regulated genes are marked on the map. **C** GO-KEGG enrichment analysis of DEGs

The top 25 functionally related genes of FDX1 in COAD (positive correlation or negative correlation) are shown in a heatmap, which showed consistently up-regulated or down-regulated (Fig. 3A). Three hundreds and sixty-four DEGs with FDX1 (|logFC|> 1, *P*_adj_ < 0.05) were selected from TCGA database using DESeq2 (336 genes were down-regulated and 28 genes were up-regulated); among the DEGs, the most down-regulated genes were GPR52, FBXO40, H4C6, C12ORF40, H4C13, etc. and the most up-regulated genes were MAGEB2, PAGE1, MRLN, APOA4, ERICH4, etc. (Fig. 3B). Gene ontology revealed that as for molecular function (MF), DEGs were involved in taste receptor activity and bitter taste receptor activity; from the perspective of cellular constituent (CC), it mainly involves in DNA packaging complex and nucleosome; in terms of biological process (BP), these DEGs were mainly involved in nucleosome assembly, sensory perception of bitter taste. KEGG enrichment analysis on the DEGs showed that the top 3 signal pathways which were significantly enriched were in taste transduction, alcoholism and systemic lupus erythematosus (Fig. 3C).

### Establishment of PPI network and detection of hub genes

**Fig. 4:**
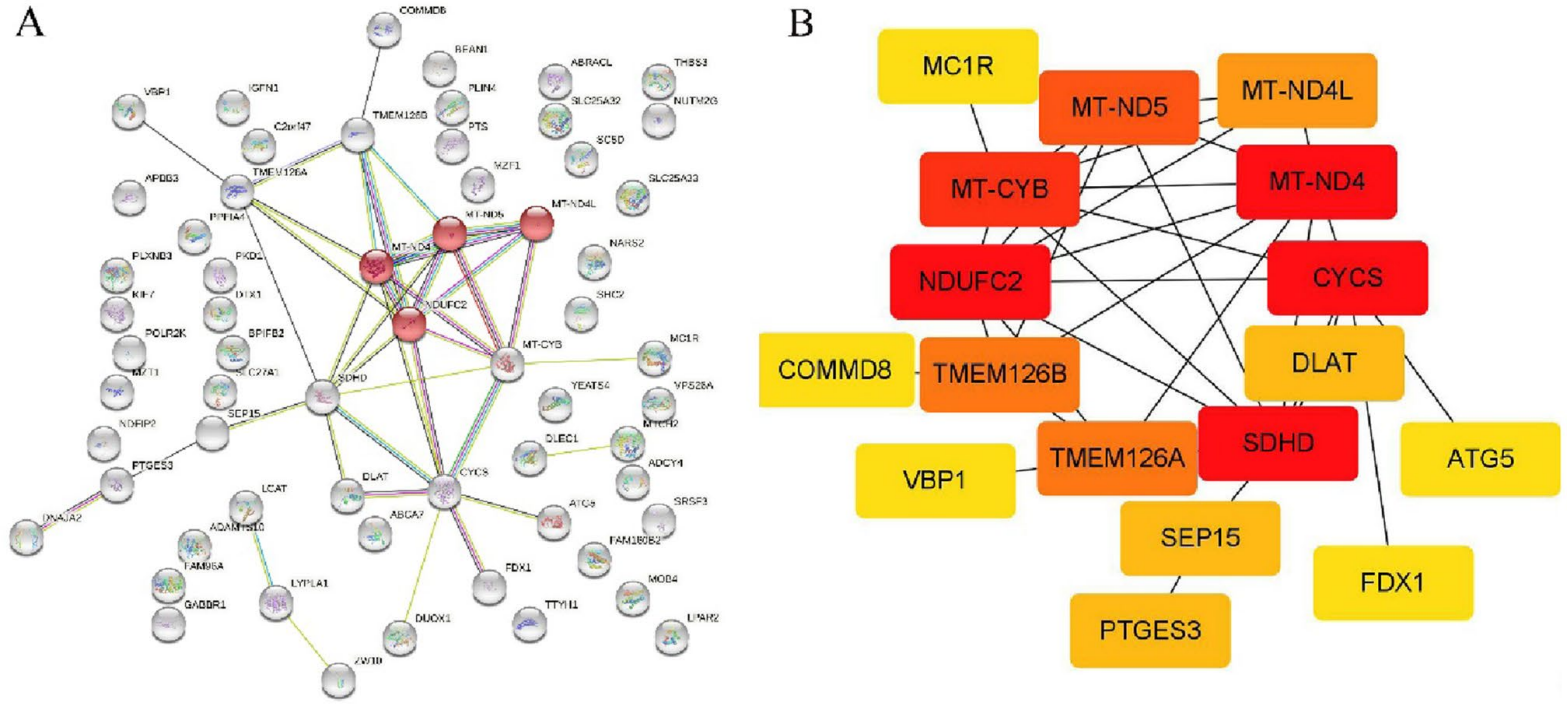
Construction of PPI network of FDX1 with its related genes and screening of hub genes. **A** PPI network diagram of FDX1 with its related genes. **B** Detection of hub genes from the PPIs network

The protein–protein interaction network between FDX1 and its related genes was constructed by STRING tool. The results showed that FDX1 only interacts with CYCS (Fig. 4A). The module analysis network contains 17 nodes and 36 edges. In the PPI network, the genes with high interaction frequency were considered as hub genes, such as CYCS, NDUFC2, MT-ND4, and SUHD (Fig. 4B).

### Relationship between expression level of FDX1 in single cell and functional status of tumor

**Fig. 5:**
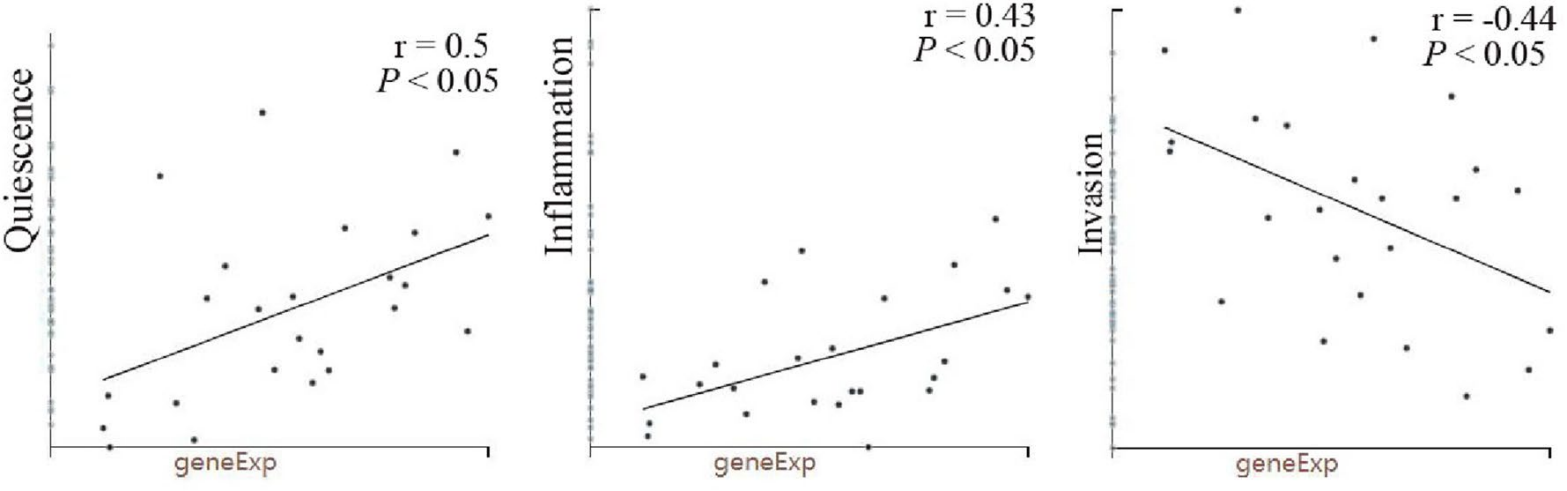
Correlation between FDX1 expression and significantly different functional states

The expression of FDX1 in single cell of COAD and its relationship with tumor functional status were analyzed via CancerSEA database. We found that FDX1 expression in COAD were significantly positively associated with “quiescence” and “inflammation” (*r* = 0.5 or 0.43, *P* < 0.05). On the contrary, FDX1 expression were negatively correlated with “invasion” (*r* = − 0.44, *P* < 0.05, Fig. 5).

### Relationship between FDX1 expression and immune cell infiltration

**Fig. 6:**
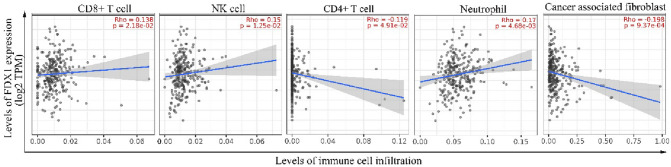
Correlation of FDX1 expression with immune cell infiltrated in COAD

The relationship between FDX1 expression and immune infiltration of COAD was analyzed via TIMER2.0. The results revealed that FDX1 expression was positively correlated with infiltration levels of CD8^+^ T cells (*r* = 0.138, *P* < 0.05), NK cells (*r* = 0.15, *P* < 0.05), and neutrophils (*r* = 0.17, *P* < 0.01). Oppositely, FDX1 expression was negatively correlated with that of CD4^+^ T cells (*r* = − 0.119, *P* < 0.05) and CAFs (*r* = − 0.198, *P* < 0.01) (Fig. 6).

### Screening of patients with high expression of FDX1 in colon cancer and characteristics of immune cell infiltration

**Fig. 7:**
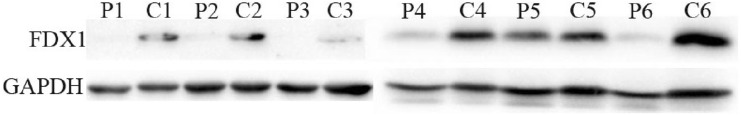
Expression levels of FDX1 were detected by Western blot analysis. P1-6: paracancerous tissues of patients 1–6, C1-6: cancer tissues of patients 1–6

**Fig. 8:**
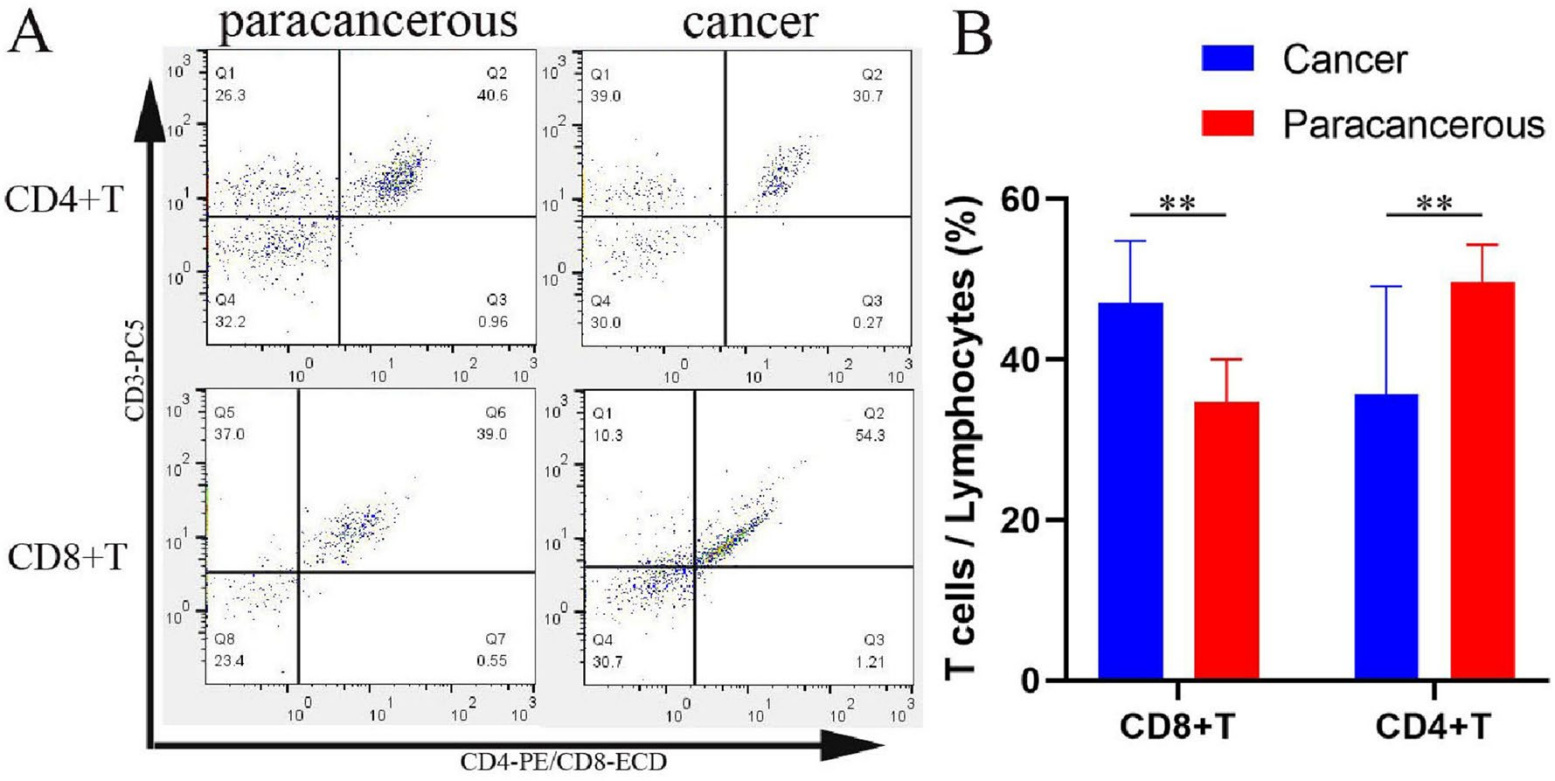
Detect CD4^+^ T and CD8^+^ T in six patients with high expression of FDX1 by flow cytometry. **A** Legend of Flow cytometry. The ratio of CD8^+^ T cells in cancer tissues was significantly higher than in paracancerous tissues but the ratio of CD4^+^ T cells was lower than in paracancerous tissues. **B** Cartogram of the proportion of CD4^+^ T and CD8^+^ T cells in cancerous and paracancerous tissues, **P < 0.01

Six patients with high expression of FDX1 in colon cancer were screened, and Western blot results showed that FDX1 expression in cancer tissues were significantly higher than that in paracancerous tissues; compared with the gray value, cancer tissue 4.78 ± 2.65 was significantly higher than paracancerous 1.0 (*P* < 0.01, Fig. 7). Flow cytometry showed that CD8^+^ T cells accounted for 47.03 ± 7.74% of the total lymphocytes in cancer tissues, significantly higher than 34.73 ± 5.27% in paracancerous tissues (*P* < 0.01, Fig. 8A). However, CD4^+^ T cells accounted for 35.70 ± 13.40% of total lymphocytes in cancer tissues, significantly lower than 49.62 ± 4.67% in paracancerous tissues (*P* < 0.01, Fig. 8B).

## Discussion

FDX1 gene encodes for iron-sulfur (Fe/S) proteins which play several roles in reduction of mitochondrial cytochrome P450 enzyme and synthesis of steroid hormones (such as pregnenolone, aldosterone and cortisol), as well as involved in the biosynthesis of heme A and Fe/S clusters (Sheftel et al. 2010; Strushkevich et al. 2011). Some studies have found that FDX1 is the direct target of elesclomol, which promotes a unique form of copper-dependent cell death, and it can improve the efficacy of targeted anticancer drugs (Tsvetkov et al. 2019). As for lung adenocarcinoma, FDX1 promotes production of ATP in tumor cells and is closely associated with glucose metabolism, fatty acid oxidation, and amino acid metabolism (Zhang et al. 2021). In addition, FDX1 is involved in the development of polycystic ovary syndrome by regulating mitochondrial and steroid metabolism (Wang et al. 2021). However, more biological activity of FDX1, as a promising therapeutic target, needs to be found in different diseases.

Analysis of FDX1 expression from TCGA database and HPA database showed that FDX1 expression was decreased in most COAD patients and increased in a few patients (Fig. 1B,* P* < 0.01). However, according to the results of K–M survival curve analysis, the survival rate of COAD patients with high FDX1 expression was significantly better than that of FDX1 low group. It has been reported that high expression of FDX1 indicates a good prognosis in Clear cell renal cell carcinoma (Bian et al. 2022). Nonetheless, why COAD patients with high expression of FDX1 have a good prognosis has not been reported. In this study, the potential mechanism of high expression of FDX1 have a good prognosis were explored based on COAD single cell function and immune infiltration level. The CancerSEA database reveals the functional status of different cancer cells at the single cell level, involving 14 functional states of 41,900 cancer single cells from 25 cancer types (Yuan et al. 2019). In this study, FDX1 expression was positively correlated with tumor cell quiescence and inflammation, and negatively correlated with tumor invasion via CancerSEA database analysis. These results suggest that high expression of FDX1 plays a crucial role in the inhibition of colon tumors. In fact, compared with traditional anticancer therapy, promoting quiescence and long-term survival has become the main strategy of tumor therapy (Recasens and Munoz 2019; Sistigu et al. 2020). Inflammation is a key component of the tumor microenvironment and a double-edged sword in tumor development. On the one hand, inflammation in the tumor microenvironment enhances tumor immunogenicity and increases susceptibility to immune regulation (O’Shaughnessy et al. 2018). On the other hand, inflammation can impair tumor immune surveillance, promote tumor cell proliferation and metastasis, as well as induce chemotherapy resistance (Buas et al. 2017; Ding et al. 2020). Previous studies suggested that FDX1 inhibits tumor invasion by regulating the expression of tumor suppressor P73 (Zhang et al. 2020). Recent evidences have shown that FDX1 is highly correlated with lipoylated proteins abundance in a variety of human tumor cells, and cell lines with high levels of lipoylated proteins are sensitive to copper-induced cell death (Tsvetkov et al. 2022). This may be another important factor that FDX1 inhibits tumor invasion.

Meanwhile, FDX1 expression was positively correlated with infiltration levels of CD8^+^ T cells, NK cells, and neutrophils but negatively correlated with that of CD4^+^ T cells and CAFs via TIMER database analysis. Studies have shown that CD8^+^ T cells and NK cells have a direct killing effect on tumors (Ben-Shaanan et al. 2018). At present, it is difficult to define the role of neutrophil in tumor microenvironment, because it not only shows the activity of promoting tumor growth and metastasis, but also has anti-tumor function (Uribe-Querol and Rosales 2015). Multiple subsets of CD4^+^ T have been shown to inhibit tumor immune response, such as Th1, Th2, and Treg cells (Fu et al. 2019). Treg cells can inhibit the proliferation and efficacy of CD8^+^ T cells, which is considered to be one of the major obstacles to successful clinical application of tumor immunotherapy (Poehlein et al. 2009; Arce Vargas et al. 2017). Cancer-associated fibroblasts (CAFs) play an important role in promoting tumor growth, metastasis and immune escape (Duperret et al. 2018; Elyada et al. 2019). These results suggest that high expression of FDX1 plays a positive role in inhibiting tumor growth and metastasis. We verified the level of CD4^+^ T cells and CD8^+^ T cells immune infiltration in 6 COAD patients with high expression of FDX1 by flow cytometry, and the results were completely consistent with the analysis of TIMER database, which effectively explained the good prognosis of patients with high expression of FDX1.

Further studies on the function of FDX1-related genes and DEGs in COAD showed consistently up-regulated or down-regulated genes, suggesting that the expression pattern of the genes was stable. Among them, GPR52, FBXO40, C12orf40, etc. were most significantly down-regulated, and MAGEB2, PAGE1, MRLN, APOA4, and ERICH4 genes were most significantly up-regulated. The study found that GPR52 downregulation involved in aggressive prostate carcinoma recurrence with MTIE (Demidenko et al. 2017); C12orf40 acted as prognostic biomarkers for kidney renal clear cell carcinoma (Yang et al. 2020). Furthermore, MAGEB2 can improve the stress tolerance of tumor cells by inhibiting the synthesis of stress granules (Lee et al. 2020); PAGE1 is also highly expressed in HCC cells and is considered as a potential biomarker and therapeutic target (Cui and Jiang 2022). GO enrichment analysis for further explored the function of DEGs and found that DEGs play important roles in molecular function, cellular composition and biological processes, such as nucleosome assembly and taste perception. KEGG enrichment analysis showed that the DEGs were significantly enriched in the taste transduction pathway. The survey found that taste impairment was more serious in COAD patients than in lung cancer and pancreatic cancer patients during diagnosis and chemotherapy (Spotten et al. 2017; Nolden et al. 2019). The results of this study support the above reports and suggest that FDX1 and its co-expressed genes play an important role in the development and progression of COAD.

Moreover, the PPI network of the DEGs was constructed. Then, hub genes were identified through module analysis of the PPI network. CYCS is the only hub gene interacting with FDX1 at high frequency. Studies have found that CYCS, as hub gene, not only plays an important role in the pathophysiology of ovarian cancer (Ha et al. 2021), but also has a vital function in bone metastasis of breast cancer and the prognosis of cervical cancer (Ma et al. 2021; Rimal et al. 2021). The high-frequency interaction between FDX1 and CYCS further solidifies its important role in the pathogenesis of COAD.

## Conclusion

In summary, we analyzed the expression of FDX1 in colon cancer by bioinformatics methods. FDX1 and its co-expressed genes play an important role in the development and progression of COAD. Moreover, high expression of FDX1 suggests a good prognosis and immune cell infiltration, which can effectively inhibit tumor cell invasion and metastasis. Regulation of FDX1 expression would be a potential therapeutic approach in inducing cuproptosis and immunotherapy for COAD.

## Data Availability

The data that support the findings of this study are available from the corresponding author on reasonable request.

## Abbreviations

COAD: Colon adenocarcinoma
BRCA: Breast invasive carcinoma
KICH: Kidney chromophobe
KIRC: Kidney renal clear cell carcinoma
LUAD: Lung adenocarcinoma
LUSC: Lung squamous cell carcinoma
PCPG: Pheochromocytoma and paraganglioma
THCA: Thyroid carcinoma
DEGs: Differentially expressed genes
